# Improving Image Quality of Chest Radiography with Artificial Intelligence-Supported Dual-Energy X-Ray Imaging System: An Observer Preference Study in Healthy Volunteers

**DOI:** 10.3390/jcm14062091

**Published:** 2025-03-19

**Authors:** Sung-Hyun Yoon, Jihang Kim, Junghoon Kim, Jong-Hyuk Lee, Ilwoong Choi, Choul-Woo Shin, Chang-Min Park

**Affiliations:** 1 Department of Radiology, Seoul National University Bundang Hospital, Seongnam-si 13620, Republic of Korea; 2 Department of Radiology, Seoul National University Hospital, Seoul National University College of Medicine, Seoul 03080, Republic of Korea; 3 Research & Development Center, DRTECH Corp., Seongnam-si 13606, Republic of Korea

**Keywords:** chest radiography, dual-energy radiography, bone suppression imaging, image quality

## Abstract

**Background/Objectives:** To compare the image quality of chest radiography with a dual-energy X-ray imaging system using AI technology (DE-AI) to that of conventional chest radiography with a standard protocol. **Methods:** In this prospective study, 52 healthy volunteers underwent dual-energy chest radiography. Images were obtained using two exposures at 60 kVp and 120 kVp, separated by a 150 ms interval. Four images were generated for each participant: a conventional image, an enhanced standard image, a soft-tissue-selective image, and a bone-selective image. A machine learning model optimized the cancellation parameters for generating soft-tissue and bone-selective images. To enhance image quality, motion artifacts were minimized using Laplacian pyramid diffeomorphic registration, while a wavelet directional cycle-consistent adversarial network (WavCycleGAN) reduced image noise. Four radiologists independently evaluated the visibility of thirteen anatomical regions (eight soft-tissue regions and five bone regions) and the overall image with a five-point scale of preference. Pooled mean values were calculated for each anatomic region through meta-analysis using a random-effects model. **Results:** Radiologists preferred DE-AI images to conventional chest radiographs in various anatomic regions. The enhanced standard image showed superior quality in 9 of 13 anatomic regions. Preference for the soft-tissue-selective image was statistically significant for three of eight anatomic regions. Preference for the bone-selective image was statistically significant for four of five anatomic regions. **Conclusions:** Images produced by DE-AI provide better visualization of thoracic structures.

## 1. Introduction

Chest radiography is one of the most widely used diagnostic imaging modalities in clinical practice. Despite being one of the oldest radiologic examinations, it remains the preferred initial diagnostic tool due to its accessibility, rapid examination, low cost, and low radiation exposure. However, interpreting chest radiographs can be challenging, as overlapping thoracic structures often create complex shadowing and reduce contrast resolution, making it difficult to distinguish anatomical details [1,2]. These limitations can obscure subtle abnormalities and hinder tissue differentiation, particularly in lung cancer screening, where chest radiography has a low detection rate for small pulmonary nodules [1,3]. The National Lung Screening Trial (NLST) found that low-dose computed tomography (CT) reduces lung cancer mortality by 20% compared to chest radiography [4,5]. Consequently, the American Cancer Society recommends low-dose CT as the preferred screening method due to its superior sensitivity [6,7].

To address the inherent limitations of chest radiography, various approaches have been explored to enhance image quality, including bone suppression, denoising, super-resolution reconstruction, and contrast enhancement [8]. Dual-energy subtraction (DES) imaging was introduced to generate tissue-selective images (typically separating soft tissue and bone), improving the visualization of lesions obscured by overlapping structures [9]. Early implementations of DES (double-exposure or single-exposure with filtered detectors) demonstrated better visualization of bone and lung lesions but faced issues like motion artifacts and noise, limiting its widespread use. Other methods have employed software-based bone suppression algorithms to similar ends [10,11,12,13]. Traditional image processing techniques for noise reduction and resolution enhancement relied on mathematical models, which offered incremental improvements [14,15].

More recently, deep learning (DL)-based methods have emerged as superior alternatives for image enhancement [16,17,18,19,20]. Data-driven algorithms can learn to suppress ribs, reduce noise, and amplify details far better than hand-crafted filters. For example, DL-based bone suppression can produce chest images with the ribs “removed,” improving lung nodule visibility [13]. Advanced generative models even allow the creation of virtual dual-energy images from a single exposure, closely approximating true low- and high-kVp image pairs without additional radiation [12]. Such AI-generated soft-tissue images have shown high fidelity (e.g., >0.98 structural similarity to actual dual-energy images) and the potential for improving pulmonary lesion detectability [12]. 

Beyond image quality, DL algorithms are increasingly assisting radiologists in interpretation tasks [21]. Recent studies have shown that comprehensive AI models can serve as effective diagnostic aids, improving radiologists’ sensitivity for detecting certain pathologies on chest X-rays [22]. In specialized applications like tuberculosis screening, AI systems have even approached or matched expert radiologist performance, achieving high sensitivity in detecting TB on chest X-rays [23]. Notably, AI can also extract novel information from X-rays; for instance, a deep learning model was able to predict patients’ lung function from a chest radiograph with accuracy comparable to spirometry [24]. This growing body of evidence underscores the transformative role AI can play in chest imaging—from improving image clarity to augmenting diagnostic performance.

Motivated by these advancements, a new chest radiography system (TruView DEXi, DRTECH Corp., Seongnam-si, Republic of Korea) integrates several AI technologies into the DES imaging workflow. This system utilizes machine learning to optimize cancellation parameters for generating soft-tissue and bone-selective images [25], employs Laplacian pyramid diffeomorphic registration to reduce motion artifacts [26], and applies a wavelet directional cycle-consistent adversarial network (WavCycleGAN) to suppress image noise [27]. It also includes a specialized algorithm to maintain optimal global and local contrast. Through this AI-supported dual-energy approach (hereafter DE-AI), the system aims to overcome prior limitations of dual-energy X-ray imaging (such as motion misregistration and noise) and produce higher-quality chest images.

In this observer preference study, we evaluated the image quality of chest radiographs acquired with the AI-supported dual-energy system compared to conventional chest X-rays. We hypothesize that the DE-AI technique, by providing clearer visualization of overlapping structures, will be preferred by radiologists.

## 2. Materials and Methods

### 2.1. Support and Funding

This study was funded by the Korea Medical Device Development Fund (Grant No. 202011B09) and received technical support from DRTECH Corporation. The control of the data and the analysis were conducted independently, without any influence from the company.

### 2.2. Participant Data

This prospective study was approved by the Institutional Review Board of our institution, and written informed consent was obtained from all participants (E-2109-708-003). We recruited only healthy volunteers for this study. A recruitment notice was posted in the hospital’s clinic and building to invite participants. Interested individuals were screened based on predefined inclusion and exclusion criteria. Participants were enrolled between 5 January 2022, and 5 February 2022, and the inclusion criteria were as follows: (1) adults over 19 years of age; and (2) those who agreed to participate in the study. In contrast, the exclusion criteria were as follows: (1) pregnant women or those who were expecting pregnancy in the near future, (2) those who were unable to provide written informed consent, and (3) individuals who had a history of thoracic disease since it may obscure the normal anatomical regions, resulting in difficulty in image quality evaluation of chest radiographs. A total of 52 participants were included in our study. There were 21 men (40.4%) and 31 women (59.6%), and the median age was 30.5 (range 23–65).

### 2.3. Image Acquisition

**Figure 1:**
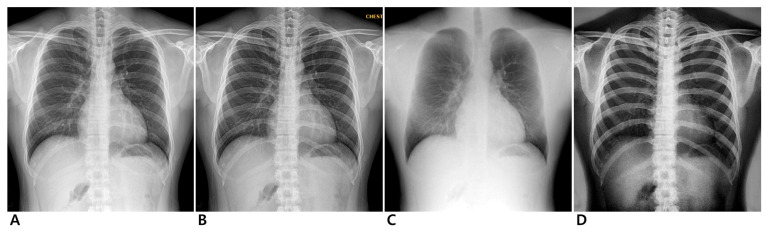
Chest radiographs of 24-year-old male participant. Conventional (**A**), enhanced standard (**B**), soft-tissue-selective (**C**), and bone-selective (**D**) images.

**Figure 2:**
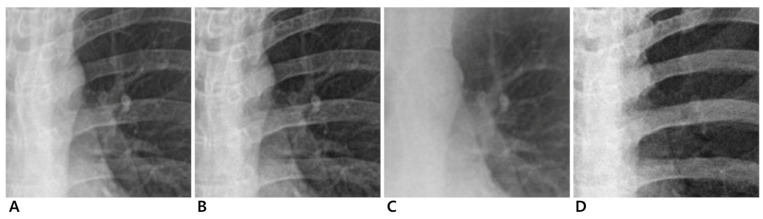
Magnified images of 24-year-old male participant. Conventional (**A**), enhanced standard (**B**), soft-tissue-selective (**C**), and bone-selective (**D**) images.

Dual-energy chest radiography was performed with a digital radiographic system (EXSYS PLUS; DRTECH Corp., Seongnam-si, Republic of Korea) equipped with a 430 × 430 mm indium-gallium-zinc oxide (IGZO) thin-film transistor detector, a CsI scintillator, and pixel dimensions of 140 micron pixel pitch. To ensure consistent positioning, all participants underwent a standard PA chest radiograph guided by a radiographer. Before the scan, participants were positioned upright with their anterior chest against the image receptor. They were instructed to place their hands on their hips with elbows slightly forward and to take a deep breath, holding it during the exposure to optimize lung expansion. The X-ray beam was directed perpendicular to the detector at approximately the level of the seventh thoracic vertebra. Images were acquired with two exposures using 60 kVp for the low energy and 120 kVp for the high energy at 150 ms intervals. Fixed tube currents of 250 mA and 200 mA were used for low- and high-energy acquisitions, respectively. To further control for variability due to body habitus, the automatic exposure control system measured X-ray transmission in real time and automatically adjusted the exposure time according to the participant’s body size (Supplemental Table S1). This ensured that all images were acquired under consistent exposure conditions, minimizing variations due to differences in participant body composition. The source-to-image receptor distance was 180 cm, and an anti-scatter grid (215 lines per inch; ratio, 10:1) was installed. The image from the high-energy exposure served as the conventional radiography image. The dual-energy subtraction algorithm generated three images for each participant: an enhanced standard image, a soft-tissue-selective image, and a bone-selective image (Figure 1 and Figure 2). All images were processed with a standardized postprocessing algorithm supplied by the manufacturer (Econsole1 SW; DRTECH Corp., Seongnam, Republic of Korea).

### 2.4. DE-AI Imaging Algorithm

DES images were generated for paired high-energy ($I_{H}$) and low-energy transmitted intensities ($I_{L}$) and the cancellation parameter (w) as follows:

(1) $$I_{Subtracted}=\exp\left(\log I_{H}-w\cdot \log I_{L}\right)$$

Cancellation parameters for soft-tissue ($w_{S}$) and bone ($w_{B}$) determine the subtracted image as a soft-tissue-selective image and bone-selective image. The DE-AI imaging system (TruviewDEXi, DRTECH Corp., Seongnam, Republic of Korea) incorporates a pre-trained machine learning model based on Extreme Gradient Boosting (XGBoost 1.4.2) to find optimal cancellation parameters [25]. The pre-trained XGBoost 1.4.2 classification model analyzes the grayscale histogram of the subtracted image and predicts the optimality of the given cancellation parameters. Laplacian pyramid diffeomorphic registration is applied to reduce motion-induced registration artifacts [26]. This deep learning-based method evaluates a pair of high-energy and low-energy images, estimates optimal motion vectors, and corrects motion artifacts using the vectors. Image noise is reduced without affecting the overall image quality using a wavelet directional cycle-consistent adversarial network (WavCycleGAN) [27]. This method preserves the high-frequency components, such as edges, and the detailed information during the noise reduction process. The architecture of WavCycleGAN is based on the cycle-consistent adversarial network (cycleGAN). The tight frame U-Net is used as the generator, and PatchGAN is used as the discriminator [28]. The model learns the mapping between the distributions of the clean and noisy images.

The DE-AI system provides enhanced standard images in addition to soft-tissue-selective images and bone-selective images. The enhanced standard imaging algorithm is a patented and unique technology distinct from other technologies (Republic of Korea, Patent No. 10-2022-0044502). Since the high- and low-energy images have different X-ray attenuation coefficients for each material, conventional subtraction in DES systems typically involves using a weighted mean of the two images to generate tissue-selective images. However, using a fixed ratio for weighting can emphasize the contrast of nonspecific materials or increase noise [29]. The enhanced standard imaging algorithm addresses this by decomposing the high-energy and the low-energy images by frequency band and selectively reconstructing images by combining frequency components to optimize contrast for specific materials. For example, it enhances low-frequency components to improve soft tissue visibility and high-frequency components to better visualize bone structures, thereby maintaining optimal global and local contrast for materials of interest.

### 2.5. Radiation Dose Estimations

Exposure parameters for each radiograph were collected. The entrance surface dose (ESD [mGy]) was calculated from the recorded exposure parameters.

### 2.6. Image Evaluation

Four radiologists (chest radiologists with 1–7 years of experience) compared the paired images independently. Paired images were compared over three sessions: (1) Conventional standard images and enhanced standard images, (2) Conventional standard images and soft-tissue selective images, and (3) Conventional standard images and bone-selective images. Before evaluation, an adaptation period allowed readers to familiarize themselves with the image viewer and the DES images. During the evaluation, the readers were blinded to the participant’s clinical information.

A total of 13 anatomical regions were included for image evaluation, which were selected and modified from previous studies [30,31]. Eight of the regions were soft-tissue regions (unobscured lung, hilum, minor fissure, heart border, retrocardiac lung, subdiaphragmatic lung, azygoesophageal recess, and proximal airway), and five of the regions were bone regions (rib, vertebral body and disc space, first costochondral joint, clavicle, and scapula). The first session evaluated 13 anatomical regions, noise reduction for soft tissue and bone, and overall appearance. Readers evaluated only soft-tissue regions in the second session and bone regions in the third session.

Image quality was graded using a five-point ordinal scale (1, strongly preferred conventional standard image; 2, moderately preferred conventional standard image; 3, no preference; 4, moderately preferred dual energy image; 5, strongly preferred dual energy image). All the images were stored in Digital Imaging and Communications in Medicine format. The window width and level of images were optimized, but readers could adjust the window width and level.

### 2.7. Statistical Analysis

A meta-analysis was conducted to pool the image quality scores provided by four readers. Meta-analysis is a statistical approach used to pool data from multiple sources to determine an overall trend while considering the variability among them. We used a random-effects model to account for the variability in scoring tendencies among the readers. The random-effects model was chosen because it allows for differences in how readers interpret image quality, considering the possibility that each reader may use slightly different criteria or thresholds. Unlike the fixed-effects model, which assumes that all readers would provide similar ratings, the random-effects model takes into account both variation within each reader’s ratings (random error) and differences between readers (true variability in scoring patterns).

Since our primary interest was to identify the overall trend in ratings rather than measure the exact agreement among readers, we did not use interobserver reliability metrics such as Cohen’s kappa or intraclass correlation coefficient (ICC). Instead, the random-effects model inherently accounts for interobserver variability by weighting each rater’s contribution inversely to their variance, thereby amplifying the influence of more consistent raters while reducing the impact of those with greater variability. This approach ensures that the pooled estimates reflect a balanced integration of observer preferences while acknowledging differences in rating consistency. Additionally, because our analysis did not involve multiple independent hypothesis tests, adjustments for multiple comparisons were not necessary.

For each anatomical region and evaluation criterion, we calculated pooled mean values and corresponding 95% confidence intervals to assess image type preference and statistical significance. The results were visualized in forest plots (Supplemental Material). Statistical analyses were performed using the ‘meta’ package in R version 4.1.2 (R Foundation for Statistical Computing, Vienna, Austria). All tests were two-tailed, and a 5% level of confidence was considered statistically significant.

## 3. Results

### 3.1. Image Acquisition and Radiation Dose Estimations

All participants successfully underwent standard PA chest radiography without any issues. No imaging failures, positioning errors, or system malfunctions occurred during dual-energy radiography acquisition or tissue-specific image generation. The mean ESD for low-energy images was 0.141 mGy (median: 0.125 mGy), and for high-energy images was 0.134 mGy (median: 0.124 mGy). The mean total ESD was 0.275 (median 0.250).

### 3.2. Image Evaluation

#### 3.2.1. Conventional Standard vs. Enhanced Standard Images

The pooled mean values and 95% confidence intervals are summarized in Table 1 and Figure S1. Preference for the enhanced standard image was statistically significant for 9 of 13 anatomic regions, noise reduction for soft tissue, noise reduction for bone, and overall appearance.

#### 3.2.2. Conventional Standard vs. Soft-Tissue-Selective Images

The pooled mean values and 95% confidence intervals are summarized in Table 2 and Figure S2. Preference for the soft-tissue selective image was statistically significant for four of eight anatomic regions, while preference for the conventional standard image was statistically significant only for azygoesophageal recess.

#### 3.2.3. Conventional Standard vs. Bone-Selective Images

The pooled mean values and 95% confidence intervals are summarized in Table 3 and Supplementary Figure S3. Preference for the bone-selective image was statistically significant for four of five anatomic regions and overall appearance.

## 4. Discussion

Our study demonstrates that integrating AI into dual-energy chest radiography can notably improve image quality. To our knowledge, while AI-driven technology and dual-energy subtraction (DES) radiography have each been used separately to enhance chest radiography image quality, research on integrating AI-driven technologies into DES radiography remains limited. The DES system employed in this study combines both approaches to complement each method, aiming to achieve significantly improved anatomical visualization compared to conventional radiography. Although deep learning (DL)-based imaging enhancement can generate synthetic bone-suppression images to improve soft tissue visualization, concerns have been raised regarding image hallucination, as it relies on data-driven image generation. Instead of synthesizing tissue-specific images using a DL-based approach, our DES system utilizes separate raw images from low- and high-kVp exposures, incorporating AI-driven technologies to extract, generate, and adjust tissue-specific images while addressing motion artifacts, noise reduction during subtraction, and optimal cancellation parameter selection. Traditional DES imaging has historically been prone to motion misregistration in dual-exposure systems and increased noise with lower signal-to-noise ratios in single-exposure systems. In contrast, radiologists using our DE-AI system did not observe noticeable motion artifacts or elevated noise levels in DE-AI images, which can be attributed to the integration of AI algorithms.

The slightly increased radiation dose for DE-AI (mean ESD, 0.28 mGy) remains within diagnostic reference levels for a PA chest X-ray [32,33] and is less than twice that of a standard single exposure [34]. In our view, this modest dose increase is justified by the diagnostic gains of DE-AI imaging—including sharper depiction of thoracic structures and improved tissue differentiation—which can aid in detecting subtle pathologies that might be missed on standard radiographs. Recent evidence supports this trade-off: higher-quality chest images, especially with bone suppression or dual-energy techniques, have been shown to improve radiologists’ ability to identify abnormalities. For example, prior studies have shown that dual-energy soft-tissue images can improve the detection of pulmonary nodules and pneumothorax compared to standard radiographs [35,36]. Moreover, incorporating AI assistance into chest X-ray interpretation has been reported to boost radiologists’ sensitivity for subtle lesions [37]. Given these findings, the enhancements provided by our DE-AI system may contribute to improved disease detection. Notably, the AI algorithms did not introduce any new artifacts; instead, they produced cleaner images that readers found preferable. This suggests that integrating AI at the image acquisition stage can yield tangible improvements in radiographic quality.

In our study, readers preferred chest radiographs from DE-AI to conventional chest radiographs across various anatomic regions. In particular, the enhanced standard image showed superior quality in most regions, which may be attributed to the algorithm’s ability to significantly improve both local and global image contrast. The image quality was not significantly different in a few regions (e.g., unobscured lung, hilum, heart border, and subdiaphragmatic lung), although the mean values in those areas still exceeded 3.0 in favor of DE-AI, indicating a slight preference trend. A relatively small number of study participants (n = 52) and images might have limited the statistical power to detect differences in those regions. Although not statistically significant in some anatomic regions, the overall preference level for soft-tissue-selective images was 3.3, indicating a mild preference over conventional images. Most bony structures in the bone-selective image were clearly visible compared to conventional images, with a significantly higher preference for rib, first costochondral joint, clavicle, and scapulae. The overall appearance of the bone-selective image was also rated favorably by the readers.

Based on our study results, even the enhanced standard image alone could markedly improve overall image quality, highlighting the potential added value of the DES radiography system. Traditionally, DES radiography systems have focused on generating tissue-specific images, such as soft-tissue-selective and bone-selective images. However, information from the two exposures can be combined into a single enhanced image that simultaneously optimizes soft tissue and bone visibility. If the DES system with a DE-AI algorithm is implemented in clinical practice, the enhanced standard image could replace the current standard image. The additional availability of soft-tissue-selective and bone-selective images could provide synergistic clinical benefits. For example, radiologists can first review the enhanced standard image for an overall assessment and then refer to tissue-specific images to further evaluate lesions of interest (e.g., examining a lung nodule on the soft-tissue image without rib interference, or confirming a bone lesion on the bone-selective image). This workflow could lead to more accurate disease diagnoses.

Our study has limitations. First, the limited number of participants may restrict the generalizability of our findings to the broader population. Since we evaluated subjective image quality preferences rather than diagnostic accuracy, standard sample size formulas were not directly applicable. Furthermore, the absence of prior data on expected differences in ratings made an a priori power calculation challenging. However, the study was designed as a preliminary evaluation of the DES system, and we observed significant differences in image quality. Nonetheless, the relatively small sample size may introduce selection bias, overestimate effect sizes, and increase interobserver variability. Future studies with larger and more diverse populations are needed to validate our findings in clinical practice. 

Second, since radiographs were obtained solely from healthy volunteers, we could not directly assess the diagnostic performance of the DES system in a clinical setting. The impact of DE-AI on detecting abnormal findings remains inferred rather than proven in this study. However, given prior evidence that dual-energy and AI enhancements can improve pathology detection, we anticipate similar benefits in a clinical setting [22,23,32,33]. Further research involving patients with thoracic abnormalities is needed to determine the extent to which DE-AI improves lesion detection rates, interpretation accuracy, and overall clinical utility. 

Third, our study did not comprehensively compare DE-AI with other AI-driven imaging enhancement methods. Although we focused on the integration of AI-driven enhancements within the DES system, future studies should evaluate its performance against existing AI-based techniques to better define its clinical significance. 

Fourth, the readers had limited prior experience with DES-based tissue-selective and enhanced standard images. Although they were provided with a brief adaptation period before the radiologic evaluation, familiarity bias may have influenced their assessments, potentially leading to more conservative results. 

Fifth, interobserver variability and reader bias remain limitations. We believe our study results account for interobserver variability, as our meta-analysis with a random-effects model mitigates the influence of inconsistent raters through inverse-variance weighting. Additionally, we assessed statistical significance by examining whether the confidence intervals remained entirely within the range indicating a significant preference, without crossing the neutrality threshold. However, the small number of readers may have allowed reader bias to disproportionately influence the overall trend. We plan to expand the reader pool in a future study with patients who have thoracic abnormalities. A larger reader pool would improve trend detection, provide a more reliable pooled effect size, and help mitigate potential reader bias by diluting individual tendencies. It would also allow for post hoc subgroup analysis to identify any systematic bias.

## 5. Conclusions

The DE-AI system produced chest radiographs with superior image quality compared to conventional chest radiography, without introducing artifacts or excessive radiation. Radiologists preferred DE-AI images for their clearer visualization of soft tissues and bony structures. While direct validation is lacking, our findings suggest that DE-AI may enhance the diagnostic value of chest PA radiographs, even in the presence of thoracic abnormalities, by helping radiologists distinguish normal anatomical structures from pathologies. We also anticipate that higher-quality images from a DE-AI system could improve computer-aided detection and diagnosis. The next step is to assess the clinical impact of these image quality gains. Prospective studies should evaluate whether DE-AI improves disease detection, such as early lung cancer and pneumonia, and enhances patient management. Additionally, examining its effect on radiologist performance and workflow is crucial, as initial findings suggest AI can expedite interpretation while maintaining accuracy.

## Figures and Tables

**Table 1:** Comparison of Conventional Standard Images with Enhanced Standard Images for 13 Anatomic Regions.

Anatomic Regions	1	2	3	4	5	Mean	95% CI
Unobscured lung	0	2	148	28	30	3.5	[2.63; 4.47]
Hilum	0	2	155	45	6	3.3	[2.94; 3.58]
Minor fissure	0	0	167	39	2	3.3	[3.14; 3.40] *
Heart border	0	3	124	65	16	3.4	[2.91; 3.98]
Retrocardiac lung	0	0	87	67	54	3.8	[3.26; 4.42] *
Subdiaphragmatic lung	0	0	111	44	53	3.7	[2.96; 4.48]
Azygoesophageal recess	0	7	79	73	49	3.8	[3.11; 4.46] *
Proximal airway	0	1	119	82	6	3.4	[3.10; 3.78] *
Noise reduction (Soft-tissue)	0	3	86	84	35	3.7	[3.08; 4.37] *
Rib	0	0	44	80	84	4.2	[3.55; 4.84] *
Vertebral body and disc space	0	0	30	95	83	4.3	[3.70; 4.82] *
First costochondral joint	0	0	106	79	23	3.6	[3.07; 4.13] *
Clavicle	0	0	65	71	72	4.4	[4.09; 4.67] *
Scapula	0	0	59	43	106	4.2	[3.41; 5.04] *
Noise reduction (Bone)	0	1	55	123	29	3.9	[3.25; 4.48] *
Overall appearance	0	3	76	83	46	3.8	[3.20; 4.45] *

Note—1 = strongly preferred conventional images, 2 = somewhat preferred conventional images, 3 = no preference, 4 = somewhat preferred enhanced images, 5 = strongly preferred enhanced images. The 95% confidence interval represents the range of the pooled mean values, not the distribution of raw scores. * The confidence interval does not contain three, indicating statistical significance.

**Table 2:** Comparison of Conventional Standard Images with Dual-Energy Soft-tissue-selective Images for Eight Anatomic Regions.

Anatomic Regions	1	2	3	4	5	Mean	95% CI
Unobscured lung	5	33	91	75	4	3.2	[2.84; 3.56]
Hilum	0	1	65	137	5	3.7	[3.50; 3.91] *
Minor fissure	0	14	181	13	0	3.0	[2.86; 3.13]
Heart border	0	18	76	99	15	3.5	[3.03; 4.05] *
Retrocardiac lung	7	76	99	26	0	2.7	[2.21; 3.17]
Subdiaphragmatic lung	9	69	111	19	0	2.7	[2.25; 3.10]
Azygoesophageal recess	10	78	102	18	0	2.6	[2.32; 2.93] *
Proximal airway	1	33	108	66	0	3.6	[3.39; 3.88] *
Overall appearance	7	51	77	73	0	3.3	[3.00; 3.58]

Note—1 = strongly preferred conventional images, 2 = somewhat preferred conventional images, 3 = no preference, 4 = somewhat preferred dual energy soft-tissue-selective images, 5 = strongly preferred dual energy soft-tissue-selective images. The 95% confidence interval represents the range of the pooled mean values, not the distribution of raw scores. * The confidence interval does not contain three, indicating statistical significance.

**Table 3:** Comparison of Conventional Standard Images with Dual-Energy Bone-selective Images for Five Anatomic Regions.

Anatomic Regions	1	2	3	4	5	Mean	95% CI
Rib	0	2	22	97	87	4.3	[3.87; 4.72] *
Vertebral body and disc space	22	39	53	75	19	3.1	[2.17; 4.12]
First costochondral joint	0	2	56	98	52	4.0	[3.40; 4.53] *
Clavicle	0	0	23	105	80	4.3	[3.77; 4.78] *
Scapula	0	1	33	84	60	4.3	[3.62; 4.92] *
Overall appearance	0	4	38	116	50	4.0	[3.47; 4.57] *

Note—1 = strongly preferred conventional images, 2 = somewhat preferred conventional images, 3 = no preference, 4 = somewhat preferred dual energy bone-selective images, 5 = strongly preferred dual energy bone-selective images. The 95% confidence interval represents the range of the pooled mean values, not the distribution of raw scores. * The confidence interval does not contain three, indicating statistical significance.

## Data Availability

The study data are not available in a public database. All author-generated code are available online and the data are available upon request (https://github.com/IWCHOI-GitHub/DE-AI_System_2023, accessed on 18 March 2025).

## Supplementary Materials

The following supporting information can be downloaded at: https://www.mdpi.com/article/10.3390/jcm14062091/s1, Figure S1. Forest plots of each item in evaluation session 1 (Conventional Standard vs. Enhanced Standard Images). Figure S2. Forest plots of each item in evaluation session 2 (Conventional Standard vs. Soft-tissue-selective Images). Figure S3. Forest plots of each item in evaluation session 3 (Conventional Standard vs. Bone-selective Images). Table S1. Acquisition parameters for chest PA radiography.
