# Supplementary material for: Improving Image Quality of Chest Radiography with Artificial Intelligence-Supported Dual-Energy X-Ray Imaging System: An Observer Preference Study in Healthy Volunteers
Source: J Clin Med. 2025 Mar 19;14(6):2091. doi: 10.3390/jcm14062091 (PMC11942644; doi:10.3390/jcm14062091)

Supplementary Materials:

Table S1. Acquisition parameters for chest PA radiography

	First				Second			
Patient	kVp	mA	msec	ESD (mGy)	kVp	mA	msec	ESD (mGy)
1	60	250	95	0.231	120	200	21	0.200
2			58	0.141			14	0.134
3			44	0.107			12	0.115
4			38	0.093			10	0.095
5			31	0.076			9	0.086
6			42	0.102			11	0.105
7			80	0.195			18	0.172
8			38	0.093			11	0.105
9			54	0.132			13	0.124
10			37	0.090			10	0.095
11			98	0.239			22	0.210
12			85	0.207			19	0.181
13			50	0.122			13	0.124
14			73	0.178			17	0.162
15			42	0.102			11	0.105
16			43	0.105			11	0.105
17			43	0.105			11	0.105
18			56	0.136			13	0.124
19			35	0.085			10	0.095
20			51	0.124			13	0.124
21			73	0.178			17	0.162
22			48	0.117			13	0.124
23			32	0.078			9	0.086
24			93	0.227			19	0.181
25			101	0.246			21	0.200
26			34	0.083			9	0.086
27			54	0.132			13	0.124
28			73	0.178			17	0.162
29			55	0.134			14	0.134
30			52	0.127			13	0.124
31			52	0.127			13	0.124
32			79	0.192			17	0.162
33			55	0.134			14	0.134
34			29	0.071			9	0.086
35			39	0.095			11	0.105
36			38	0.093			11	0.105
37			55	0.134			14	0.134
38			82	0.200			19	0.181
39			44	0.107			12	0.115
40			51	0.124			13	0.124
41			43	0.105			12	0.115
42			69	0.168			17	0.162
43			29	0.071			8	0.076
44			131	0.319			26	0.248
45			148	0.361			29	0.277
46			36	0.088			10	0.095
47			44	0.107			12	0.115
48			56	0.136			13	0.124
49			49	0.119			12	0.115
50			48	0.117			12	0.115
51			53	0.129			14	0.134
52			73	0.178			17	0.162
Mean			58	0.141			14	0.134
Median			52	0.125			13	0.124

Note—ESD, entrance surface dose

Figure S1. Forest plots of each item in evaluation session 1 (Conventional Standard vs. Enhanced Standard Images)

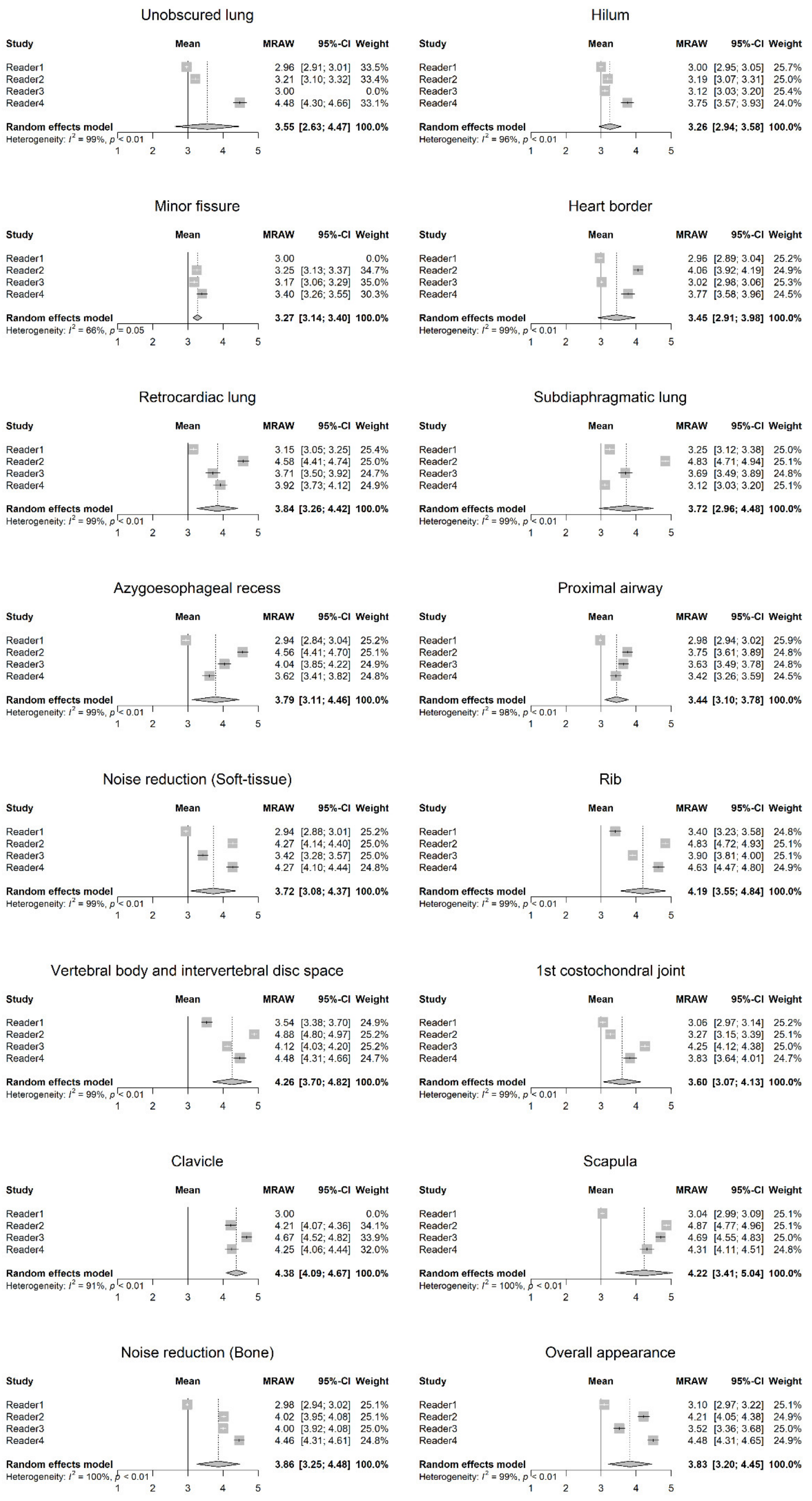

Figure S2. Forest plots of each item in evaluation session 2 (Conventional Standard vs. Soft-tissue Images)

Unobscured lung

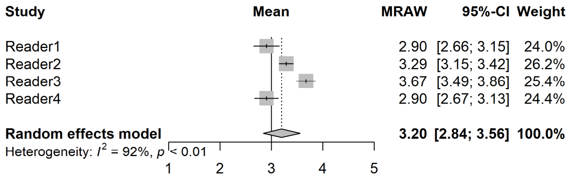

Hilum

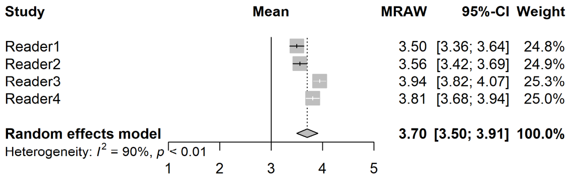

Minor fissure

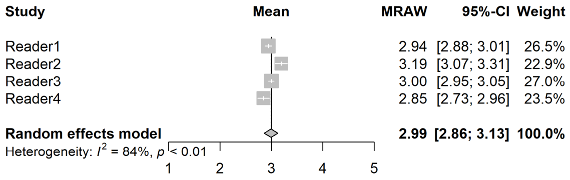

Heart border

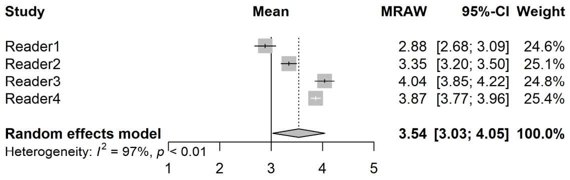

Retrocardiac lung

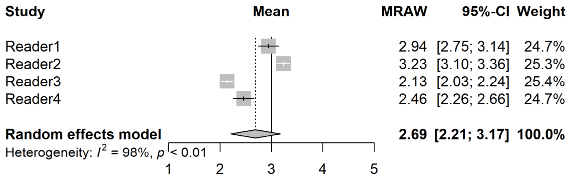

Subdiaphragmatic lung

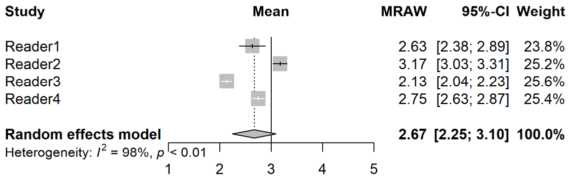

Azygoesophageal recess

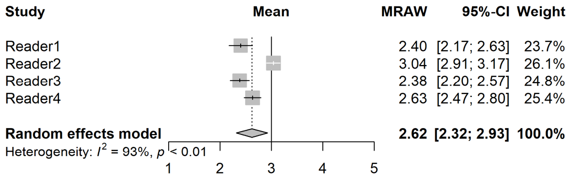

Proximal airway

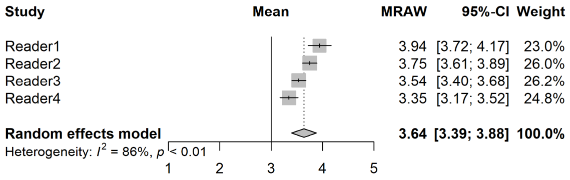

Overall appearance

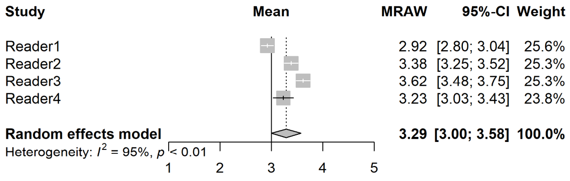

Figure S3. Forest plots of each item in evaluation session 3 (Conventional Standard vs. Bone Images)

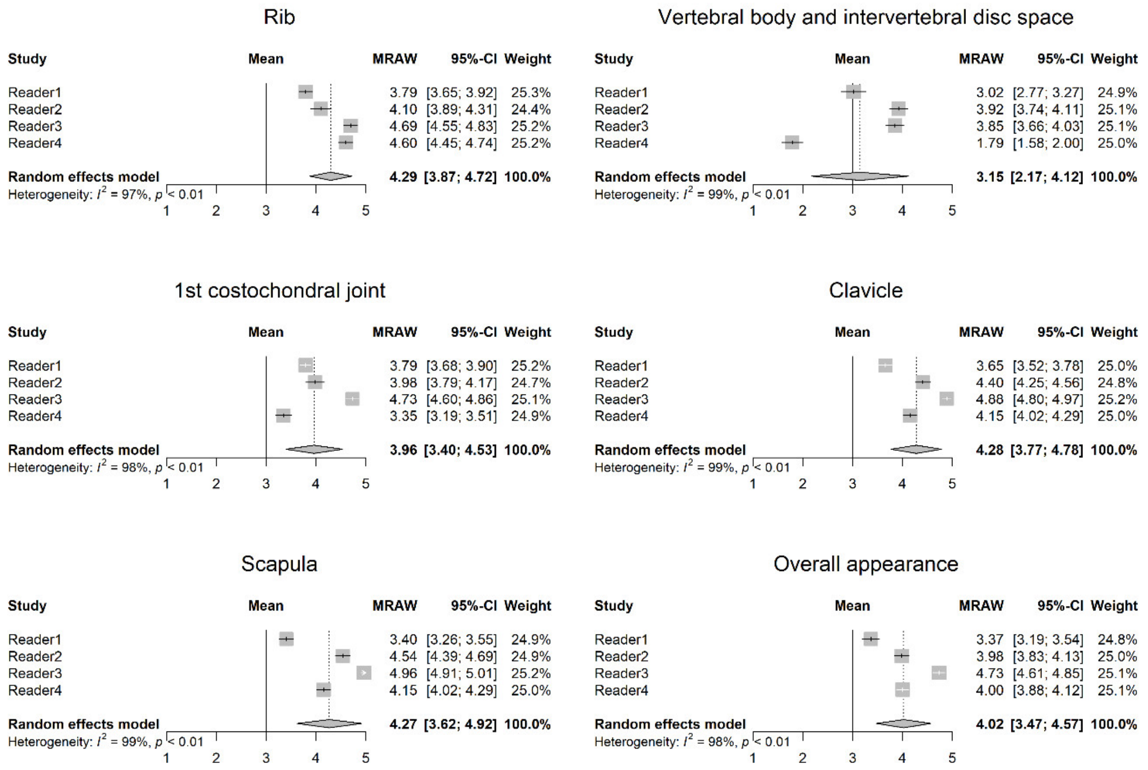

Supplement: Supplementary file 1 [file jcm-14-02091-s001.zip › jcm-3453735-supplementary.pdf]
